# Soft *ω*-regular open sets and soft nearly Lindelöfness

**DOI:** 10.1016/j.heliyon.2022.e09954

**Published:** 2022-07-18

**Authors:** Samer Al Ghour

**Affiliations:** Department of Mathematics and Statistics, Jordan University of Science and Technology, Irbid 22110, Jordan

**Keywords:** Soft regular open, Soft semi regular, Soft almost regular, Soft nearly Lindelöf, Soft generated soft topological space, Soft induced topological spaces

## Abstract

In this paper, we define the notion of “soft *ω*-regular openness,” which lies exactly between “soft regular openness” and “soft *ω*-openness.” Through soft *ω*-regular open sets, we introduce the notions of soft semi *ω*-regularity as a weaker form of soft semi regularity and soft almost *ω*-regularity as a strong form of soft almost regularity. We prove that soft *ω*-regular open sets of a soft topological space form a soft topology. Also, we prove that soft semi *ω*-regularity and soft almost *ω*-regularity are independent notions. In addition, we reveal the relationships between soft topology and classical (parametric) topology. Finally, we characterize soft nearly Lindelöfness and improve several results related to soft nearly Lindelöfness using the concept of soft *ω*-regular open sets.

## 1 Introduction and preliminaries

Many real-life problems have their own uncertainties. Accordingly, it is not possible to deal with these problems by traditional methods. For more than thirty years, fuzzy set theory [1], rough set theory [2], and vague set theory [3] have played an essential role in dealing with these problems. Molodostov [4] states that each of these mathematical structures has its drawbacks. The research via soft sets has entered in most scientific fields. Soft set theory has been applied to solve problems using Riemann integral, Beron's integral, game theory, function smoothness, operations research, measure theory, probability, and decision-making problems [4], [5], [6].

Shabir and Naz [7] initiated soft topology. Since that time, the generalization of topological concepts in soft topology has become the focus of many researchers, such as soft compact and soft Lindelöf [8], [9], [10], [11], [12], soft connected [13], soft paracompact [13], soft extremely disconnected [14], soft separable spaces [15], soft separation axioms [16], [17], [18], soft metric spaces [19], [20], [21], and soft homogeneous spaces [22], [23]. The field of research in soft topology is still active (see for example [24], [25], [26], [27], [28], [29], [30], [31], [32], [33], [34], [35], [36], [37]), and substantial contributions can still be made.

Closure and interior operators were used to give rise to several different new classes of sets, while a few others are the so-called regular sets. Researchers have discovered applications for these regular sets not only in mathematics but even in a variety of fields outside of mathematics [38], [39], [40].

The targets of this paper are to scrutinize the behaviors of soft *ω*-regular open sets via soft topological spaces, to characterize soft nearly Lindelöfness, to improve several results related to soft nearly Lindelöfness using the concept of soft *ω*-regular open sets, and to open the door to redefine and investigate some of the soft topological concepts such as soft compactness, soft menger, soft separation axioms, and soft continuity, via soft *ω*-regular open sets.

The arrangement of this article is as follows:

In Section 2, we define soft *ω*-regular open sets. We investigate the properties of these soft sets and point out their relationships with some famous generalized soft open sets. We also show the relationships between soft *ω*-regular open sets in soft topology and their general topological counterparts.

In Section 3, we define two new classes of soft separation axioms: soft semi *ω*-regular and soft almost *ω*-regular. We investigate their properties and point out their relationships with some famous soft separation axioms. We also show the relationships between each of them in soft topology and their general topological counterparts.

In Section 4, we characterize soft nearly Lindelöfness and improve several results related to soft nearly Lindelöfness using the concept of soft *ω*-regular open sets.

In Section 5, we give some conclusions and possible future work.

Definition 1.1 [4] Let *Z* be a universal set and *E* be a set of parameters. A soft set over *Z* relative to *E* is a function G:E⟶P(Z). The collection of all soft sets over *Z* relative to *E* will be denoted by SS(Z,E).

Definition 1.2 Let K∈SS(Z,E).

(a) [22] If $K(e)=\left\{ \begin{array}{cc} Y\; & \text{if }e=a\\ \emptyset \; & \text{if }e\neq a \end{array}\right.$, then *K* will be denoted by $a_{Y}$.

(b) [22] If K(e)=Y for all e∈E, then *K* will be denoted by $C_{Y}$.

(c) [19] If $K(e)=\left\{ \begin{array}{cc} \left\{z\right\}\; & \text{if }e=a\\ \emptyset \; & \text{if }e\neq a \end{array}\right.$, then *K* will be denoted by $a_{z}$ and will be called a soft point of SS(Z,E). The set of all soft points of SS(Z,E) will be denoted by SP(Z,E).

Definition 1.3 For any SS(Z,E),

(a) [41] $C_{\emptyset }$ is said to be the null soft set and will be denoted by $0_{E}$.

(b) [41] $C_{Z}$ is said to be the absolute soft set and will be denoted by $1_{E}$.

(c) [19] K∈SS(Z,E) is said to be a countable soft set of SS(Z,E) if K(e) is a countable set for each e∈E. The collection of all countable soft sets of SS(Z,E) will be denoted by CSS(Z,E).

Definition 1.4 [41] Let M,N∈SS(Z,E). Then

(a) *M* is said to be a soft subset of *N*, denoted by $M\overset{˜}{\subseteq }N$, if M(e)⊆N(e) for every e∈E.

(b) the soft union of *M* and *N* is denoted by $M\overset{˜}{\cup }N$ and defined to be the soft set $M\overset{˜}{\cup }N\in SS(Z,E)$ where $\left(M\overset{˜}{\cup }N\right)\left(e\right)=M(e)\cup N\left(e\right)$ for every e∈E.

(c) the soft intersection of *M* and *N* is denoted by $M\overset{˜}{\cap }N$ and defined to be the soft set $M\overset{˜}{\cap }N\in SS(Z,E)$ where $\left(M\overset{˜}{\cap }N\right)\left(e\right)=M(e)\cap N\left(e\right)$ for every e∈E.

(d) the difference of *M* and *N* is denoted by M−N and defined to be the soft set M−N∈SS(Z,E) where (M−N)(e)=M(e)−N(e) for every e∈E.

Definition 1.5 [42] Let $\left\{S_{\lambda }:\lambda \in \Gamma \right\}\subseteq SS(Z,E)$. Then

(a) the soft union of these soft sets is the soft set denoted by $\overset{˜}{\cup_{\lambda \in \Gamma }}S_{\lambda }$ and defined by $\left(\overset{˜}{\cup_{\lambda \in \Gamma }}S_{\lambda }\right)\left(e\right)=\cup_{\lambda \in \Gamma }S_{\lambda }\left(e\right)$ for all e∈E.

(b) the soft intersection of these soft sets is the soft set denoted by $\overset{˜}{\cap_{\lambda \in \Gamma }}S_{\lambda }$ and defined by $\left(\overset{˜}{\cap_{\lambda \in \Gamma }}S_{\lambda }\right)\left(e\right)=\cap_{\lambda \in \Gamma }S_{\lambda }\left(e\right)$ for all e∈E.

Definition 1.6 [19] Let M∈SS(Z,E) and $a_{z}\in SP(Z,E)$. Then $a_{z}$ is said to belongs to *M* ($a_{z}\overset{˜}{\in }M$) if z∈M(a).

Definition 1.7 [7] A collection *δ* ⊆ SS(Z,E) is said to be a soft topology on *Z* relative to *E* if

(a) $0_{E},1_{E}\in \delta$,

(b) *δ* is closed under arbitrary soft union,

(c) *δ* is closed under finite soft intersection.

If *δ* is a soft topology on *Z* relative to *E*, then the triplet (Z,δ,E) is said to be a soft topological space, the members of *δ* are called soft open sets in (Z,δ,E) and their soft complements are called soft closed sets in (Z,δ,E). The family of all soft closed sets in (Z,δ,E) will be denoted by $\delta^{c}$.

From now on, topological space and soft topological space will be denoted by TS and STS, respectively. Let (Z,ρ) be a TS, (Z,δ,E) be a STS, U⊆Z, and K∈SS(Z,E). Then the interior of *U* in (Z,ρ), the closure of *U* in (Z,ρ), the soft interior of *K* in (Z,δ,E), and the closure of *K* in (Z,δ,E) will be denoted by $Int_{\rho }(U)$, $Cl_{\rho }(U)$, $Int_{\delta }(K)$, and $Cl_{\delta }(K)$, respectively.

Definition 1.8 [43] A subset *U* of a TS (Z,ρ) is said to be a regular open set in (Z,ρ) if $U=Int_{\rho }(Cl_{\rho }(U))$. The complement of a regular open set in (Z,ρ) is said to be a regular closed set.

The family of all soft regular open sets (resp. regular closed sets) of a TS (Z,ρ) is denoted by RO(Z,ρ) (resp. RC(Z,ρ)).

Definition 1.9 [44] A subset *W* of a TS (Z,ρ) is said to be an *ω*-regular open set in (Z,ρ) if for any z∈W, we find U∈RO(Z,ρ) such that z∈U and U−W is countable. The complement of an *ω*-regular open set in (Z,ρ) is said to be an *ω*-regular closed set.

The family of all *ω*-regular open sets (resp. *ω*-regular closed sets) of a TS (Z,ρ) is denoted by ωRO(Z,ρ) (resp. ωRC(Z,ρ)).

Definition 1.10 A TS (Y,γ) is said to be

(a) [45] semi regular if for every y∈Y and every S∈γ such that y∈S, we find T∈RO(Y,γ) such that y∈T⊆S.

(b) [46] almost regular if for every y∈Y and every S∈RO(Y,γ) such that y∈S, we find T∈γ such that $y\in T\subseteq Cl_{\gamma }(T)\subseteq S$.

(c) [44] semi *ω*-regular if for every y∈Y and every S∈γ such that y∈S, we find T∈ωRO(Y,γ) such that y∈T⊆S.

(d) [44] almost *ω*-regular if for every y∈Y and every S∈ωRO(Y,γ) such that y∈S, we find T∈γ such that $y\in T\subseteq Cl_{\gamma }(T)\subseteq S$.

Definition 1.11 [47] A soft set *S* of a STS (Z,δ,E) is said to be a soft regular open set in (Z,δ,E) if $S=Int_{\delta }(Cl_{\delta }(S))$. The soft complement of a soft regular open set in (Z,δ,E) is said to be a soft regular closed set.

The collection of all soft regular open sets (resp. soft regular closed sets) of a STS (Z,δ,E) is denoted by RO(Z,δ,E) (resp. RC(Z,δ,E)).

Definition 1.12 A STS (Z,δ,E) is said to be

(a) [48] soft regular if for every $e_{z}\in SP(Z,E)$ and every F∈δ such that $e_{z}\overset{˜}{\in }F$, we find H∈δ such that $e_{z}\overset{˜}{\in }H\overset{˜}{\subseteq }Cl_{\delta }(H)\overset{˜}{\subseteq }F$.

(b) [49] soft semi regular if for every $e_{z}\in SP(Z,E)$ and every F∈δ such that $e_{z}\overset{˜}{\in }F$, we find H∈RO(Z,δ,E) such that $e_{z}\overset{˜}{\in }H\overset{˜}{\subseteq }F$.

(c) [50] soft almost regular if for every $e_{z}\in SP(Z,E)$ and every F∈RO(Z,δ,E) such that $e_{z}\overset{˜}{\in }F$, we find H∈δ such that $e_{z}\overset{˜}{\in }H\overset{˜}{\subseteq }Cl_{\delta }(H)\overset{˜}{\subseteq }F$.

Definition 1.13 [51] Let (Z,δ,E) be a STS and let K∈SS(Z,E). Then

(a) (Z,δ,E) is said to be soft nearly Lindelöf if every soft cover of $1_{E}$ from RO(Z,δ,E) has a countable subcover.

(b) *K* is said to be soft nearly Lindelöf relative to (Z,δ,E) if every soft cover of *K* from RO(Z,δ,E) has a countable subcover.

Theorem 1.14 *[7]* *For any STS* (Z,δ,E) *and any* e∈E *,* {G(e):G∈δ} *is a topology on Z. This topology is denoted by* $\delta_{e}$ *.*

Theorem 1.15 *Let* (Z,ρ) *be a TS and E be a set of parameters. Then*

*(a)* *[52]* {G∈SS(Z,E):G(e)∈ρfor everye∈E} *is a soft topology on Z relative to E. This soft topology is denoted by* τ(ρ) *.*

*(b)* *[22]* $\left\{C_{V}:V\in \rho \right\}$ *is a soft topology on Z relative to E. This soft topology will be denoted by* C(ρ) *.*

It is not difficult to check that the two types of soft topology in Theorem 1.15 are extended soft topologies [53].

Theorem 1.16 *[22]* *For any collection of TSs* $\left\{\left(Z,\rho_{e}\right):e\in E\right\}$ *,* $\left\{G\in SS\left(Z,E\right):G\left(e\right)\in \rho_{e}\;\text{for every}\;e\in E\right\}$ *is a soft topology on Z relative to E. This soft topology will be denoted by* $\underset{e\in E}{⊕}\rho_{e}$ *.*

Theorem 1.17 *[32]* *For any STS* (Z,δ,E) *,* $\left\{M\in SS(Z,E):\;\text{for every}e_{z}\overset{˜}{\in }M,\;\text{we find}\;H\in \delta \;\text{such that}\;e_{z}\overset{˜}{\in }H\;\text{and}\;H-M\in CSS(Z,E)\right\}$ *is a soft topology on Z relative to E. This soft topology is denoted by* $\delta_{\omega }$ *.*

A non-empty subset K⊆SS(Z,E) is said to have the countable soft intersection property if for any countable subfamily $K_{1}\subseteq K$, we have $\overset{˜}{\cap_{K\in K_{1}}}K\neq 0_{E}$.

## 2 Soft *ω*-regular open sets

In this section, we define soft *ω*-regular open sets. We investigate the properties of these soft sets and point out their relationships with some famous generalized soft open sets. We also show the relationships between soft *ω*-regular open sets in soft topology and their general topological counterparts.

Definition 2.1 A soft set *S* of a STS (Z,δ,E) is said to be a soft *ω*-regular open set in (Z,δ,E) if for any $e_{z}\overset{˜}{\in }S$, there exists R∈RO(Z,δ,E) such that $e_{z}\overset{˜}{\in }R$ and R−S ∈CSS(Z,E). The soft complement of a soft *ω*-regular open set in (Z,δ,E) is said to be soft *ω*-regular closed set.

The family of all soft *ω*-regular open sets (resp. soft *ω*-regular closed sets) of a STS (Z,δ,E) is denoted by ωRO(Z,δ,E) (resp. ωRC(Z,δ,E)).

Theorem 2.2 *For any STS* (Z,δ,E) *,* RO(Z,δ,E)⊆ωRO(Z,δ,E) *.*

Proof Straightforward. □

Theorem 2.3 *For any STS* (Z,δ,E) *,* {G−K:G∈RO(Z,δ,E)andK∈CSS(Z,E)}⊆ωRO(Z,δ,E) *.*

Proof Let G∈RO(Z,δ,E) and K∈CSS(Z,E). Let $e_{z}\overset{˜}{\in }G-K$. Then we have $e_{z}\overset{˜}{\in }G\in$ RO(Z,δ,E) and G−(G−K)=K∈CSS(Z,E). Hence, G−K∈ωRO(Z,δ,E). □

The following example shows that the inclusion in Theorem 2.2 cannot be replaced by equality, in general:

Example 2.4 Let Z=R, E=Z, *ρ* be the usual topology on *Z*, and δ={L∈SS(Z,E):L(e)∈ρ for every e∈E}. Since $1_{E}\in RO(Z,\delta ,E)$ and $C_{Q}\in CSS(Z,E)$, then by Theorem 2.3, $C_{Q^{c}}=1_{E}-C_{Q}\in \omega RO(Z,\delta ,E)$. On the other hand, $C_{Q^{c}}\notin RO(Z,\delta ,E)$.

Theorem 2.5 *For any STS* (Z,δ,E) *,* $\omega RO(Z,\delta ,E)\subseteq \delta_{\omega }$ *.*

Proof Let S∈ωRO(Z,δ,E) and let $e_{z}\overset{˜}{\in }S$. Then there exists R∈RO(Z,δ,E)⊆δ such that $e_{z}\overset{˜}{\in }R$ and R−S ∈CSS(Z,E). Therefore, $S\in \delta_{\omega }$. □

Inclusion in Theorem 2.5 cannot be replaced by equality in general, as the following example clarifies:

Example 2.6 Let Z=R, E={a,b}, and $\delta =\left\{0_{E},1_{E},C_{\left(-\infty ,1\right]}\right\}$. Then $C_{\left(-\infty ,1\right]}\in \delta \subseteq \delta_{\omega }$. If $C_{\left(-\infty ,1\right]}\in \omega RO(Z,\delta ,E)$, then there exists G∈RO(Z,δ,E) such that $a_{1}\overset{˜}{\in }G$ and $G-C_{\left(-\infty ,1\right]}$ ∈CSS(Z,E). Since $Int_{\delta }\left(Cl_{\delta }\left(C_{\left(-\infty ,1\right]}\right)\right)=Int_{\delta }\left(1_{E}\right)=1_{E}\neq C_{\left(-\infty ,1\right]}$, then $C_{\left(-\infty ,1\right]}\notin RO(Z,\delta ,E)$. Thus, $G=1_{E}$. As a result, $1_{E}-C_{\left(-\infty ,1\right]}=C_{\left(1,\infty \right)}$ ∈CSS(Z,E) which is not correct. Therefore, $C_{\left(-\infty ,1\right]}\notin \omega RO(Z,\delta ,E)$.

Theorem 2.7 *For any STS* (Z,δ,E) *,* (Z,ωRO(Z,δ,E),E) *is a STS.*

Proof (1) We have $0_{E},1_{E}\in \omega RO(Z,\delta ,E)$.

(2) Let S,T∈ωRO(Z,δ,E) and let $e_{z}\overset{˜}{\in }S\overset{˜}{\cap }T$. Then $e_{z}\overset{˜}{\in }S$ and $e_{z}\overset{˜}{\in }T$. So, there exist M,N∈RO(Z,δ,E) such that $e_{z}\overset{˜}{\in }M\overset{˜}{\cap }N$ and M−S,N−T ∈CSS(Z,E). Now,

$\begin{array}{ccc} \left(M\overset{˜}{\cap }N\right)-\left(S\overset{˜}{\cap }T\right)\; & =\; & \left(M\overset{˜}{\cap }N\right)\overset{˜}{\cap }\left(1_{E}-\left(S\overset{˜}{\cap }T\right)\right)\\ \; & =\; & \left(M\overset{˜}{\cap }N\right)\overset{˜}{\cap }\left(\left(1_{E}-S\right)\overset{˜}{\cup }\left(1_{E}-T\right)\right)\\ \; & \overset{˜}{\subseteq }\; & \left(M\overset{˜}{\cap }\left(1_{E}-S\right)\right)\overset{˜}{\cup }\left(N\overset{˜}{\cap }\left(1_{E}-T\right)\right)\\ \; & =\; & \left(M-S\right)\overset{˜}{\cup }\left(N-T\right)\text{.} \end{array}$

Since M−S,N−T ∈CSS(Z,E), then $\left(M-S\right)\overset{˜}{\cup }\left(N-T\right)\in CSS(Z,E)$, and so $\left(M\overset{˜}{\cap }N\right)-\left(S\overset{˜}{\cap }T\right)\in CSS(Z,E)$. Also, $M\overset{˜}{\cap }N\in RO(Z,\delta ,E)$. Hence, $S\overset{˜}{\cap }T\in$ ωRO(Z,δ,E).

(3) Let $\left\{S_{\alpha }:\alpha \in \Delta \right\}\subseteq \omega RO(Z,\delta ,E)$. Let $e_{z}\overset{˜}{\in }\overset{˜}{\cup_{\alpha \in \Delta }}S_{\alpha }$. Choose β∈Δ such that $e_{z}\overset{˜}{\in }S_{\beta }$. Then there exists R∈RO(Z,δ,E) such that $e_{z}\overset{˜}{\in }R$ and $R-S_{\beta }$ ∈CSS(Z,E). Now,

$$R-\left(\overset{˜}{\cup_{\alpha \in \Delta }}S_{\alpha }\right)=\overset{˜}{\cap \alpha \in \Delta }\left(R-S_{\alpha }\right)\overset{˜}{\subseteq }R-S_{\beta }\text{.}$$

Since $R-S_{\beta }$ ∈CSS(Z,E), then $R-\left(\overset{˜}{\cup_{\alpha \in \Delta }}S_{\alpha }\right)\in CSS(Z,E)$. Hence, $\overset{˜}{\cup_{\alpha \in \Delta }}S_{\alpha }\in \omega RO(Z,\delta ,E)$. □

Theorem 2.8 *Let* (Z,δ,E) *be a STS and* S∈SS(Z,E) *. Then* S∈ωRO(Z,δ,E) *if and only if for every* $e_{z}\overset{˜}{\in }S$ *, there exists* R∈RO(Z,δ,E) *and* K∈CSS(Z,E) *such that* $e_{z}\overset{˜}{\in }R$ *and* $R-K\overset{˜}{\subseteq }S$ *.*

Proof *Necessity*. Suppose that S∈ωRO(Z,δ,E) and let $e_{z}\overset{˜}{\in }S$. Then there exists R∈RO(Z,δ,E) such that $e_{z}\overset{˜}{\in }R$ and R−S∈CSS(Z,E). Put $K=R-S=R\overset{˜}{\cap }\left(1_{E}-S\right)$. Then K∈CSS(Z,E) and

$$R-K=R\overset{˜}{\cap }\left(1_{E}-K\right)=R\overset{˜}{\cap }\left(1_{E}-\left(R\overset{˜}{\cap }\left(1_{E}-S\right)\right)\right)=R\overset{˜}{\cap }\left(\left(1_{E}-R\right)\overset{˜}{\cup }S\right)=R\overset{˜}{\cap }S\overset{˜}{\subseteq }S\text{.}$$

*Sufficiency*. Suppose that for every $e_{z}\overset{˜}{\in }S$, there exists R∈RO(Z,δ,E) and K∈CSS(Z,E) such that $e_{z}\overset{˜}{\in }R$ and $R-K\overset{˜}{\subseteq }S$. Let $e_{z}\overset{˜}{\in }S$. Then by assumption, there exists R∈RO(Z,δ,E) and K∈CSS(Z,E) such that $e_{z}\overset{˜}{\in }R$ and $R-K\overset{˜}{\subseteq }S$. So, we have $R-S\overset{˜}{\subseteq }K\in CSS(Z,E)$, and hence, R−S∈CSS(Z,E). It follows that S∈ωRO(Z,δ,E). □

Proposition 2.9 *Let* (Z,δ,E) *be a STS such that for some* e∈E *,* F(e)≠∅ *for every* $F\in \delta -\left\{0_{E}\right\}$ *. Then*

*(a) For each* G∈δ *,* $Cl_{\delta_{e}}(G(e))=\left(Cl_{\delta }(G)\right)(e)$ *.*

*(b) For each* $N\in \delta^{c}$ *,* $Int_{\delta_{e}}(N(e))=\left(Int_{\delta }(N)\right)(e)$ *.*

*(c) For each* G∈δ *,* $\left(Int_{\delta }(Cl_{\delta }(G))\right)(e)=Int_{\delta_{e}}(Cl_{\delta_{e}}(G(e)))$ *.*

Proof (a) Let G∈δ. Then by Proposition 7 of [7], $Cl_{\delta_{e}}(G(e))\subseteq \left(Cl_{\delta }(G)\right)(e)$. To see that $\left(Cl_{\delta }(G)\right)(e)\subseteq Cl_{\delta_{e}}(G(e))$, suppose to the contrary that there exists $z\in \left(Cl_{\delta }(G)\right)(e)-Cl_{\delta_{e}}(G(e))$. Then $e_{z}\overset{˜}{\in }Cl_{\delta }(G)$ and $z\notin Cl_{\delta_{e}}(G(e))$. Since $z\notin Cl_{\delta_{e}}(G(e))$, then there exists $U\in \delta_{e}$ such that z∈U and U∩G(e)=∅. Choose M∈δ such that M(e)=U. Then we have $e_{z}\overset{˜}{\in }Cl_{\delta }(G)\overset{˜}{\cap }M$, and hence $G\overset{˜}{\cap }M\neq 0_{E}$. Since $G\overset{˜}{\cap }M\in \delta -\left\{0_{E}\right\}$, then by assumption, $\left(G\overset{˜}{\cap }M\right)(e)\neq \emptyset$. But $\left(G\overset{˜}{\cap }M\right)(e)=U\cap G(e)=\emptyset$, which is a contradiction.

(b) Let $N\in \delta^{c}$. Then $1_{E}-N\in \delta$. So, by (a), $Cl_{\delta_{e}}(\left(1_{E}-N\right)(e))=\left(Cl_{\delta }(\left(1_{E}-N\right))\right)(e)$. And thus,

$$Z-Cl_{\delta_{e}}(\left(1_{E}-N\right)(e))=Z-\left(Cl_{\delta }\left(1_{E}-N\right)\right)(e))\text{.}$$

But $Z-Cl_{\delta_{e}}(\left(1_{E}-N\right)(e))=Z-Cl_{\delta_{e}}\left(Z-N\left(e\right)\right)=Int_{\delta_{e}}(N(e))$, and

$Z-\left(Cl_{\delta }\left(1_{E}-N\right)\right)(e))=\left(1_{E}-Cl_{\delta }\left(1_{E}-N\right)\right)(e)=\left(Int_{\delta }(N)\right)(e)$. Therefore, $Int_{\delta_{e}}(N(e))=\left(Int_{\delta }(N)\right)(e)$.

(c) Let G∈δ. Since $Cl_{\delta }(G)\in \delta^{c}$, then by (b), $\left(Int_{\delta }(Cl_{\delta }(G))\right)(e)=Int_{\delta_{e}}(\left(Cl_{\delta }(G)\right)(e))$. Since G∈δ, then by (a), $\left(Cl_{\delta }(G)\right)(e)=Cl_{\delta_{e}}(G(e))$. Thus,

$$\left(Int_{\delta }(Cl_{\delta }(G))\right)(e)=Int_{\delta_{e}}(\left(Cl_{\delta }(G)\right)(e))=Int_{\delta_{e}}(Cl_{\delta_{e}}(G(e)))\text{.}$$

 □

Theorem 2.10 *Let* (Z,δ,E) *be a STS such that for some* e∈E *,* F(e)≠∅ *for every* $F\in \delta -\left\{0_{E}\right\}$ *. Then for every* G∈RO(Z,δ,E) *,* $G(e)\in RO(Z,\delta_{e})$ *.*

Proof Let G∈RO(Z,δ,E). Then $G=Int_{\delta }(Cl_{\delta }(G))$. So, by Proposition 2.9 (c),

$$G(e)=\left(Int_{\delta }(Cl_{\delta }(G))\right)(e)=Int_{\delta_{e}}(Cl_{\delta_{e}}(G(e)))\text{.}$$

Hence, $G(e)\in RO(Z,\delta_{e})$. □

Theorem 2.11 *Let* (Z,δ,E) *be a STS such that* F(e)≠∅ *for all* $F\in \delta -\left\{0_{E}\right\}$ *and* e∈E *and let* G∈δ *. Then* G∈RO(Z,δ,E) *if and only if* $G(e)\in RO(Z,\delta_{e})$ *for every* e∈E *.*

Proof *Necessity*. Suppose that G∈RO(Z,δ,E) and let e∈E. Then by Theorem 2.10, $G(e)\in RO(Z,\delta_{e})$ for every e∈E.

*Sufficiency*. Suppose that $G(e)\in RO(Z,\delta_{e})$ for every e∈E. Then $G(e)=Int_{\delta_{e}}(Cl_{\delta_{e}}(G(e)))$ for every e∈E. So, by Proposition 2.9 (c), $G(e)=\left(Int_{\delta }(Cl_{\delta }(G))\right)(e)$ for every e∈E and hence, $G=Int_{\delta }(Cl_{\delta }(G))$. It follows that G∈RO(Z,δ,E). □

Corollary 2.12 *Let* (Z,ρ) *be a TS and E be any set of parameters. Let* W∈P(Z)−{∅} *. Then* W∈RO(Z,ρ) *if and only if* $C_{W}\in RO(Z,C\left(\rho \right),E)$ *.*

Theorem 2.13 *Let* (Z,δ,E) *be a STS such that for some* e∈E *,* F(e)≠∅ *for every* $F\in \delta -\left\{0_{E}\right\}$ *. Then for every* G∈ωRO(Z,δ,E) *,* $G(e)\in \omega RO(Z,\delta_{e})$ *.*

Proof Let G∈ωRO(Z,δ,E) and let z∈G(e). Then $e_{z}\overset{˜}{\in }G$ and so there exists R∈RO(Z,δ,E) such that $e_{z}\overset{˜}{\in }R$ and R−G∈CSS(Z,E). So, we have z∈R(e) and by Theorem 2.10, $R(e)\in RO(Z,\delta_{e})$. Also, since R−G∈CSS(Z,E), then R(e)−G(e)=(R−G)(e) is a countable subset of *Z*. Therefore, $G(e)\in \omega RO(Z,\delta_{e})$. □

Theorem 2.14 *Let* (Z,δ,E) *be a STS such that* F(e)≠∅ *for all* $F\in \delta -\left\{0_{E}\right\}$ *and* e∈E *, and let* G∈δ *. If* G∈ωRO(Z,δ,E) *, then* $G(e)\in \omega RO(Z,\delta_{e})$ *for every* e∈E *.*

Proof Suppose that G∈ωRO(Z,δ,E) and let e∈E. Then by Theorem 2.13, $G(e)\in \omega RO(Z,\delta_{e})$ for every e∈E. □

Question 2.15 Let (Z,δ,E) be a STS such that F(e)≠∅ for all $F\in \delta -\left\{0_{E}\right\}$ and e∈E, and let G∈δ such that G(e)∈ωRO(Z,δ,E) for every e∈E. Is it true that G∈ωRO(Z,δ,E)?

The following theorem gives a partial answer for Question 2.15:

Theorem 2.16 *Let* (Z,ρ) *be a TS and E be any set of parameters. Let* W∈P(Z)−{∅} *. Then* W∈ωRO(Z,ρ) *if and only if* $C_{W}\in \omega RO(Z,C\left(\rho \right),E)$ *.*

Proof *Necessity*. Suppose that W∈ωRO(Z,ρ) and let $e_{z}\overset{˜}{\in }C_{W}$. Then z∈W and so, there exists U∈RO(Z,ρ) such that z∈U and U−W is countable. So, we have $e_{z}\overset{˜}{\in }C_{U}$ and by Corollary 2.12, $C_{U}\in$ RO(Z,C(ρ),E). Moreover, $C_{U}-C_{W}=C_{U-W}\in CSS(Z,E)$. Hence, $C_{W}\in \omega RO(Z,C\left(\rho \right),E)$.

*Sufficiency*. Let $C_{W}\in \omega RO(Z,C\left(\rho \right),E)$. Choose e∈E. Then by Theorem 2.14, $W=\left(C_{W}\right)(e)\in \omega RO(Z,\rho )$. □

Theorem 2.17 *Let* $\left\{\left(Z,\rho_{e}\right):e\in E\right\}$ *be a collection of TSs. Then* $G\in \omega RO(Z,{⊕}_{e\in E}\rho_{e},E)$ *if and only if* $G(e)\in \omega RO(Z,\rho_{e})$ *for all* e∈E *.*

Proof *Necessity*. Suppose that $G\in \omega RO(Z,{⊕}_{e\in E}\rho_{e},E)$ and let e∈E. Let z∈G(e). Then $e_{z}\overset{˜}{\in }G$. So, there exists $R\in RO(Z,{⊕}_{e\in E}\rho_{e},E)$ such that $e_{z}\overset{˜}{\in }R$ and R−G∈CSS(Z,E). By Proposition 3.28 of [54], $R(e)\in RO(Z,\rho_{e})$. Moreover, since R−G∈CSS(Z,E), then R(e)−G(e)=(R−G)(e) is countable. Therefore, $G(e)\in \omega RO(Z,\rho_{e})$.

*Sufficiency*. Suppose that $G(e)\in \omega RO(Z,\rho_{e})$ for all e∈E. Let $e_{z}\overset{˜}{\in }G$. Then z∈G(e)∈ $\omega RO(Z,\rho_{e})$. So, there exists $U\in RO(Z,\rho_{e})$ such that z∈U and U−G(e) is countable. Now, we have $e_{z}\overset{˜}{\in }e_{U}$ and by Proposition 3.28 of [54], $e_{U}\in RO(Z,{⊕}_{e\in E}\rho_{e},E)$. Moreover, $e_{U}-G\in CSS(Z,E)$. Therefore, $G\in \omega RO(Z,{⊕}_{e\in E}\rho_{e},E)$. □

Corollary 2.18 *Let* (Z,ρ) *be a TS and E be any set of parameters. Let* G∈SS(Z,E) *. Then* G∈ωRO(Z,τ(ρ),E) *if and only if* G(e)∈ωRO(Z,ρ) *for every* e∈E *.*

## 3 Soft semi *ω*-regularity and soft almost *ω*-regularity

In this section, we define two new classes of soft separation axioms: soft semi *ω*-regular and soft almost *ω*-regular. We investigate their properties and point out their relationships with some famous soft separation axioms. We also show the relationships between each of them in soft topology and their general topological counterparts.

Definition 3.1 A STS (Z,δ,E) is said to be soft semi *ω*-regular if for each $e_{z}\in SP(Z,E)$ and each F∈δ such that $e_{z}\overset{˜}{\in }F$, there exists G∈ωRO(Z,δ,E) such that $e_{z}\overset{˜}{\in }G\overset{˜}{\subseteq }F$.

Theorem 3.2 *Every soft semi regular STS is soft semi ω-regular.*

Proof Follows from the definitions and Theorem 2.2. □

Soft semi *ω*-regularity does not imply soft semi regularity in general:

Example 3.3 Let Z={1,2,3,4}, E={a,b}, and

$\delta =\left\{C_{U}:U\in \left\{\emptyset ,Z,\left\{1\right\},\left\{2\right\},\left\{1,2\right\},\left\{1,2,3\right\}\right\}\right\}$. It is easy to see that $RO(Z,\delta ,E)=\left\{C_{U}:U\in \left\{\emptyset ,Z,\left\{1\right\},\left\{2\right\}\right\}\right\}$. So, by Theorem 2.3, ωRO(Z,δ,E)=SS(Z,E). Hence, (Z,δ,E) is soft semi *ω*-regular. On the other hand, since $a_{3}\overset{˜}{\in }C_{\left\{1,2,3\right\}}\in \delta$ but there is no H∈RO(Z,δ,E) such that $a_{3}\overset{˜}{\in }H\overset{˜}{\subseteq }C_{\left\{1,2,3\right\}}$, then (Z,δ,E) is not soft semi regular.

Theorem 3.4 *Let* (Z,δ,E) *be a soft semi regular STS such that for some* e∈E *,* F(e)≠∅ *for every* $F\in \delta -\left\{0_{E}\right\}$ *. Then* $\left(Z,\delta_{e}\right)$ *is semi regular.*

Proof Let z∈Z and $U\in \delta_{e}$ such that z∈U. Choose F∈δ such that F(e)=U. Then we have $e_{z}\overset{˜}{\in }F\in \delta$. Since (Z,δ,E) is soft semi regular, then there exists G∈RO(Z,δ,E) such that $e_{z}\overset{˜}{\in }G\overset{˜}{\subseteq }F$. By Theorem 2.10, $G(e)\in RO(Z,\delta_{e})$. Also, we have z∈G(e)⊆F(e)=U. It follows that $\left(Z,\delta_{e}\right)$ is semi regular. □

Corollary 3.5 *If* (Z,δ,E) *is a soft semi regular STS such that* F(e)≠∅ *for all* $F\in \delta -\left\{0_{E}\right\}$ *and* e∈E *, then* $\left(Z,\delta_{e}\right)$ *is semi regular for every* e∈E *.*

Question 3.6 Let (Z,δ,E) be a STS such that F(e)≠∅ for all $F\in \delta -\left\{0_{E}\right\}$ and e∈E, and $\left(Z,\delta_{e}\right)$ is semi regular for all e∈E. Is it true that (Z,δ,E) is soft semi regular?

The following theorem gives a partial answer for Question 3.6:

Theorem 3.7 *Let* (Z,ρ) *be a TS and E be any set of parameters. Then* (Z,C(ρ),E) *is soft semi regular if and only if* (Z,ρ) *is semi regular.*

Proof *Necessity*. Suppose that (Z,C(ρ),E) is soft semi regular. Choose e∈E. Then by Theorem 3.4, $\left(Z,{\left(C\left(\rho \right)\right)}_{e}\right)$ is semi regular. But ${\left(C\left(\rho \right)\right)}_{e}=\rho$. Hence, (Z,ρ) is semi regular.

*Sufficiency*. Suppose that (Z,ρ) is semi regular. Let $C_{U}\in C\left(\rho \right)$ and let $e_{z}\overset{˜}{\in }C_{U}$. Then z∈U∈ρ. Since (Z,ρ) is semi regular, then there exists V∈RO(Z,ρ) such that z∈V⊆U. Thus, $e_{z}\overset{˜}{\in }C_{V}\overset{˜}{\subseteq }C_{U}$ and by Corollary 2.12, $C_{V}\in RO(Z,C\left(\rho \right),E)$. Therefore, (Z,C(ρ),E) is soft semi regular. □

Theorem 3.8 *Let* $\left\{\left(Z,\rho_{e}\right):e\in E\right\}$ *be a collection of TSs. Then* $\left(Z,{⊕}_{e\in E}\rho_{e},E\right)$ *is soft semi regular if and only if* $\left(Z,\rho_{e}\right)$ *is semi regular for all* e∈E *.*

Proof *Necessity*. Suppose that $\left(Z,{⊕}_{e\in E}\rho_{e},E\right)$ is soft semi regular and let e∈E. Let z∈Z and $U\in \rho_{e}$ such that z∈U. Then $e_{z}\overset{˜}{\in }e_{U}\in {⊕}_{e\in E}\rho_{e}$. Since $\left(Z,{⊕}_{e\in E}\rho_{e},E\right)$ is soft semi regular, then there exists $G\in RO(Z,{⊕}_{e\in E}\rho_{e},E)$ such that $e_{z}\overset{˜}{\in }G\overset{˜}{\subseteq }e_{U}$. So, we have z∈G(e)⊆U. Also, by Proposition 3.28 of [54], $G(e)\in RO(Z,\rho_{e})$. Hence, $\left(Z,\rho_{e}\right)$ is semi regular.

*Sufficiency*. Suppose that $\left(Z,\rho_{e}\right)$ is semi regular for all e∈E. Let $e_{z}\in SP(Z,E)$ and $F\in {⊕}_{e\in E}\rho_{e}$ such that $e_{z}\overset{˜}{\in }F$. Then z∈F(e)∈ $\rho_{e}$. Since $\left(Z,\rho_{e}\right)$ is semi regular, then there exists $V\in RO(Z,\rho_{e})$ such that z∈V⊆F(e). Thus, we have $e_{z}\overset{˜}{\in }e_{V}\overset{˜}{\subseteq }F$ and by Proposition 3.28 of [54], $e_{V}\in RO(Z,{⊕}_{e\in E}\rho_{e},E)$. It follows that $\left(Z,{⊕}_{e\in E}\rho_{e},E\right)$ is soft semi regular. □

Corollary 3.9 *Let* (Z,ρ) *be a TS and E be any set of parameters. Then* (Z,τ(ρ),E) *is soft semi regular if and only if* (Z,ρ) *is semi regular.*

Theorem 3.10 *Let* (Z,δ,E) *be a soft semi ω-regular STS such that for some* e∈E *,* F(e)≠∅ *for every* $F\in \delta -\left\{0_{E}\right\}$ *. Then* $\left(Z,\delta_{e}\right)$ *is semi ω-regular.*

Proof Let z∈Z and $U\in \delta_{e}$ such that z∈U. Choose F∈δ such that F(e)=U. Then we have $e_{z}\overset{˜}{\in }F\in \delta$. Since (Z,δ,E) is soft semi *ω*-regular, then there exists G∈ωRO(Z,δ,E) such that $e_{z}\overset{˜}{\in }G\overset{˜}{\subseteq }F$. By Theorem 2.13, $G(e)\in \omega RO(Z,\delta_{e})$. Also, we have z∈G(e)⊆F(e)=U. It follows that $\left(Z,\delta_{e}\right)$ is semi *ω*-regular. □

Corollary 3.11 *If* (Z,δ,E) *is a soft semi ω-regular STS such that* F(e)≠∅ *for all* $F\in \delta -\left\{0_{E}\right\}$ *and* e∈E *, then* $\left(Z,\delta_{e}\right)$ *is semi ω-regular for every* e∈E *.*

Question 3.12 Let (Z,δ,E) be a STS such that F(e)≠∅ for all $F\in \delta -\left\{0_{E}\right\}$ and e∈E, and $\left(Z,\delta_{e}\right)$ is semi *ω*-regular for all e∈E. Is it true that (Z,δ,E) is soft semi *ω*-regular?

The following theorem gives a partial answer for Question 3.12:

Theorem 3.13 *Let* (Z,ρ) *be a TS and E be any set of parameters. Then* (Z,C(ρ),E) *is soft semi ω-regular if and only if* (Z,ρ) *is semi ω-regular.*

Proof *Necessity*. Suppose that (Z,C(ρ),E) is soft semi *ω*-regular. Choose e∈E. Then by Theorem 3.10, $\left(Z,{\left(C\left(\rho \right)\right)}_{e}\right)$ is semi *ω*-regular. But ${\left(C\left(\rho \right)\right)}_{e}=\rho$. Hence, (Z,ρ) is semi *ω*-regular.

*Sufficiency*. Suppose that (Z,ρ) is semi *ω*-regular. Let $C_{U}\in C\left(\rho \right)$ where U∈ρ, and let $e_{z}\overset{˜}{\in }C_{U}$. Then z∈U∈ρ. Since (Z,ρ) is semi *ω*-regular, then there exists V∈ωRO(Z,ρ) such that z∈V⊆U. So, $e_{z}\overset{˜}{\in }C_{V}\overset{˜}{\subseteq }C_{U}$. Also, by Theorem 2.16, $C_{V}\in \omega RO(Z,C\left(\rho \right),E)$. It follows that (Z,C(ρ),E) is soft semi *ω*-regular. □

Theorem 3.14 *Let* $\left\{\left(Z,\rho_{e}\right):e\in E\right\}$ *be a collection of TSs. Then* $\left(Z,{⊕}_{e\in E}\rho_{e},E\right)$ *is soft semi ω-regular if and only if* $\left(Z,\rho_{e}\right)$ *is semi ω-regular for all* e∈E *.*

Proof *Necessity*. Suppose that $\left(Z,{⊕}_{e\in E}\rho_{e},E\right)$ is soft semi *ω*-regular and let e∈E. Let z∈Z and $U\in \rho_{e}$ such that z∈U. Then $e_{z}\overset{˜}{\in }e_{U}\in {⊕}_{e\in E}\rho_{e}$. Since $\left(Z,{⊕}_{e\in E}\rho_{e},E\right)$ is soft semi *ω*-regular, then there exists $G\in \omega RO(Z,{⊕}_{e\in E}\rho_{e},E)$ such that $e_{z}\overset{˜}{\in }G\overset{˜}{\subseteq }e_{U}$. Thus, we have z∈G(e)⊆U. Also, by Theorem 2.17, $G(e)\in \omega RO(Z,\rho_{e})$. Hence, $\left(Z,\rho_{e}\right)$ is semi *ω*-regular.

*Sufficiency*. Suppose $\left(Z,\rho_{e}\right)$ is semi *ω*-regular for all e∈E. Let $e_{z}\in SP(Z,E)$ and $F\in {⊕}_{e\in E}\rho_{e}$ such that $e_{z}\overset{˜}{\in }F$. Then z∈F(e)∈ $\rho_{e}$. Since $\left(Z,\rho_{e}\right)$ is semi *ω*-regular, then there exists $V\in \omega RO(Z,\rho_{e})$ such that z∈V⊆F(e). Thus, we have $e_{z}\overset{˜}{\in }e_{V}\overset{˜}{\subseteq }F$ and by Theorem 2.17, $e_{V}\in \omega RO(Z,{⊕}_{e\in E}\rho_{e},E)$. It follows that $\left(Z,{⊕}_{e\in E}\rho_{e},E\right)$ is soft semi *ω*-regular. □

Corollary 3.15 *Let* (Z,ρ) *be a TS and E be any set of parameters. Then* (Z,τ(ρ),E) *is soft semi ω-regular if and only if* (Z,ρ) *is semi ω-regular.*

Definition 3.16 A STS (Z,δ,E) is said to be soft almost *ω*-regular if for each $e_{z}\in SP(Z,E)$ and each F∈ωRO(Z,δ,E) such that $e_{z}\overset{˜}{\in }F$, there exists G∈δ such that $e_{z}\overset{˜}{\in }G\overset{˜}{\subseteq }Cl_{\delta }(G)\overset{˜}{\subseteq }F$.

Theorem 3.17 *Every soft almost ω-regular STS is soft almost regular.*

Proof Follows from the definitions and Theorem 2.2. □

The following two examples will show that the converse of Theorem 3.17 need not be true, and that soft semi *ω*-regularity and soft almost *ω*-regularity are independent concepts:

Example 3.18 Let Z={1,2,3}, E={a,b}, and $\delta =\left\{0_{E},1_{E},C_{\left\{1\right\}},C_{\left\{2,3\right\}}\right\}$. Then (Z,δ,E) is soft almost regular and soft semi regular. On the other hand, since $b_{2}\in SP(Z,E)$ and $C_{\left\{2\right\}}\in \omega RO(Z,\delta ,E)$ with $b_{2}\overset{˜}{\in }C_{\left\{2\right\}}$ but there is not G∈δ such that $b_{2}\overset{˜}{\in }G\overset{˜}{\subseteq }Cl_{\delta }(G)\overset{˜}{\subseteq }C_{\left\{2\right\}}$, then (Z,δ,E) is not soft almost *ω*-regular.

Example 3.19 Let (Z,δ,E) be as in Example 2.6. It is not difficult to check that $\omega RO(Z,\delta ,E)=\left\{0_{E},1_{E}\right\}$. Then clearly that (Z,δ,E) is soft almost *ω*-regular. On the other hand, since $a_{1}\overset{˜}{\in }C_{\left(-\infty ,1\right]}$ but there is not $G\in \omega RO(Z,\delta ,E)=\left\{0_{E},1_{E}\right\}$ such that $a_{1}\overset{˜}{\in }G\overset{˜}{\subseteq }C_{\left(-\infty ,1\right]}$, then (Z,δ,E) is not soft semi *ω*-regular.

Theorem 3.20 *Every soft semi ω-regular soft almost ω-regular is soft regular.*

Proof Let (Z,δ,E) be soft semi *ω*-regular and soft almost *ω*-regular. Let $e_{z}\in SP(Z,E)$ and F∈δ such that $e_{z}\overset{˜}{\in }F$. Since (Z,δ,E) is soft semi *ω*-regular, then there exists G∈ωRO(Z,δ,E) such that $e_{z}\overset{˜}{\in }G\overset{˜}{\subseteq }F$. Since (Z,δ,E) is soft almost *ω*-regular, then there exists K∈δ such that $e_{z}\overset{˜}{\in }K\overset{˜}{\subseteq }Cl_{\delta }(K)\overset{˜}{\subseteq }G\overset{˜}{\subseteq }F$. Therefore, (Z,δ,E) is soft regular. □

Theorem 3.21 *Let* (Z,ρ) *be a TS and E be any set of parameters. Then* (Z,C(ρ),E) *is soft almost regular if and only if* (Z,ρ) *is almost regular.*

Proof *Necessity*. Suppose that (Z,C(ρ),E) is soft almost regular. Let z∈Z and U∈RO(Z,ρ) such that z∈U. By Corollary 2.12, $C_{U}\in RO(Z,C\left(\rho \right),E)$. Pick e∈E. Since (Z,C(ρ),E) is soft almost regular and $e_{z}\overset{˜}{\in }C_{U}\in RO(Z,C\left(\rho \right),E)$, then there exists V∈ρ such that $e_{z}\overset{˜}{\in }C_{V}\overset{˜}{\subseteq }Cl_{C\left(\rho \right)}(C_{V})=C_{Cl_{\rho }(V)}\overset{˜}{\subseteq }C_{U}$. Thus, $z\in V\subseteq Cl_{\rho }(V)\subseteq U$. Hence, (Z,ρ) is almost regular.

*Sufficiency*. Suppose that (Z,ρ) is almost regular. Let $e_{z}\in SP(Z,E)$ and $C_{U}\in RO(Z,C\left(\rho \right),E)$ such that $e_{z}\overset{˜}{\in }C_{U}$. Then by Corollary 2.12, U∈RO(Z,ρ). Since (Z,ρ) is almost regular and z∈U∈RO(Z,ρ), there exists V∈ρ such that $z\in V\subseteq Cl_{\rho }(V)=U$. Thus, we have $C_{V}\in C\left(\rho \right)$ and $e_{z}\overset{˜}{\in }C_{V}\overset{˜}{\subseteq }C_{Cl_{\rho }(V)}=Cl_{C\left(\rho \right)}(C_{V})\overset{˜}{\subseteq }C_{U}$. Hence, (Z,C(ρ),E) is soft almost regular. □

Theorem 3.22 *Let* (Z,ρ) *be a TS and E be any set of parameters. Then* (Z,C(ρ),E) *is soft almost ω-regular if and only if* (Z,ρ) *is almost ω-regular.*

Proof *Necessity*. Suppose that (Z,C(ρ),E) is soft almost *ω*-regular. Let z∈Z and U∈ωRO(Z,ρ) such that z∈U. By Theorem 2.16, $C_{U}\in \omega RO(Z,C\left(\rho \right),E)$. Pick e∈E. Since (Z,C(ρ),E) is soft almost *ω*-regular and $e_{z}\overset{˜}{\in }C_{U}\in \omega RO(Z,C\left(\rho \right),E)$, then there exists V∈ρ such that $e_{z}\overset{˜}{\in }C_{V}\overset{˜}{\subseteq }Cl_{C\left(\rho \right)}(C_{V})=C_{Cl_{\rho }(V)}\overset{˜}{\subseteq }C_{U}$. Thus, $z\in V\subseteq Cl_{\rho }(V)\subseteq U$. Hence, (Z,ρ) is almost *ω*-regular.

*Sufficiency*. Suppose that (Z,ρ) is almost *ω*-regular. Let $e_{z}\in SP(Z,E)$ and $C_{U}\in \omega RO(Z,C\left(\rho \right),E)$ such that $e_{z}\overset{˜}{\in }C_{U}$. Then by Theorem 2.16, U∈ωRO(Z,ρ). Since (Z,ρ) is almost *ω*-regular and z∈U∈ωRO(Z,ρ), there exists V∈ρ such that $z\in V\subseteq Cl_{\rho }(V)=U$. Thus, we have $C_{V}\in C\left(\rho \right)$ and $e_{z}\overset{˜}{\in }C_{V}\overset{˜}{\subseteq }C_{Cl_{\rho }(V)}=Cl_{C\left(\rho \right)}(C_{V})\;\overset{˜}{\subseteq }C_{U}$. Hence, (Z,C(ρ),E) is soft almost *ω*-regular. □

Theorem 3.23 *Let* $\left\{\left(Z,\rho_{e}\right):e\in E\right\}$ *be a collection of TSs. Then* $\left(Z,{⊕}_{e\in E}\rho_{e},E\right)$ *is soft almost regular if and only if* $\left(Z,\rho_{e}\right)$ *is almost regular for all* e∈E *.*

Proof *Necessity*. Suppose that $\left(Z,{⊕}_{e\in E}\rho_{e},E\right)$ is soft almost regular and let e∈E. Let z∈Z and $U\in RO(Z,\rho_{e})$ such that z∈U. Then $e_{z}\overset{˜}{\in }e_{U}$ and by Proposition 3.28 of [54], $e_{U}\in RO(Z,{⊕}_{e\in E}\rho_{e},E)$. Since $\left(Z,{⊕}_{e\in E}\rho_{e},E\right)$ is soft almost regular, then there exists $G\in {⊕}_{e\in E}\rho_{e}$ such that $e_{z}\overset{˜}{\in }G\overset{˜}{\subseteq }Cl_{{⊕}_{e\in E}\rho_{e}}(G)\overset{˜}{\subseteq }e_{U}$. Thus, we have $G(e)\in \rho_{e}$ and z∈G(e) $\subseteq \left(Cl_{{⊕}_{e\in E}\rho_{e}}(G)\right)(e)\subseteq U$. Also, by Lemma 4.9 of [55], $\left(Cl_{{⊕}_{e\in E}\rho_{e}}(G)\right)(e)=Cl_{\rho_{e}}(G(e))$. Hence, $\left(Z,\rho_{e}\right)$ is almost regular.

*Sufficiency*. Suppose that $\left(Z,\rho_{e}\right)$ is almost regular for all e∈E. Let $e_{z}\in SP(Z,E)$ and $F\in RO(Z,{⊕}_{e\in E}\rho_{e},E)$ such that $e_{z}\overset{˜}{\in }F$. Then by Proposition 3.28 of [54], $F(e)\in RO(Z,\rho_{e})$. Since $\left(Z,\rho_{e}\right)$ is almost regular and z∈F(e)∈ $RO(Z,\rho_{e})$, then there exists $V\in \rho_{e}$ such that $z\in V\subseteq Cl_{\rho_{e}}(V)\subseteq F(e)$. Now, we have $e_{V}\in {⊕}_{e\in E}\rho_{e}$ and $e_{z}\overset{˜}{\in }e_{V}\overset{˜}{\subseteq }e_{Cl_{\rho_{e}}(V)}=Cl_{{⊕}_{e\in E}\rho_{e}}(e_{V})\overset{˜}{\subseteq }F$. It follows that $\left(Z,{⊕}_{e\in E}\rho_{e},E\right)$ is soft almost regular. □

Corollary 3.24 *Let* (Z,ρ) *be a TS and E be any set of parameters. Then* (Z,τ(ρ),E) *is soft almost regular if and only if* (Z,ρ) *is almost regular.*

Theorem 3.25 *Let* $\left\{\left(Z,\rho_{e}\right):e\in E\right\}$ *be a collection of TSs. Then* $\left(Z,{⊕}_{e\in E}\rho_{e},E\right)$ *is soft almost ω-regular if and only if* $\left(Z,\rho_{e}\right)$ *is almost ω-regular for all* e∈E *.*

Proof *Necessity*. Suppose that $\left(Z,{⊕}_{e\in E}\rho_{e},E\right)$ is soft almost *ω*-regular and let e∈E. Let z∈Z and $U\in \omega RO(Z,\rho_{e})$ such that z∈U. Then $e_{z}\overset{˜}{\in }e_{U}$ and by Theorem 2.17, $e_{U}\in \omega RO(Z,{⊕}_{e\in E}\rho_{e},E)$. Since $\left(Z,{⊕}_{e\in E}\rho_{e},E\right)$ is soft almost *ω*-regular, then there exists $G\in {⊕}_{e\in E}\rho_{e}$ such that $e_{z}\overset{˜}{\in }G\overset{˜}{\subseteq }Cl_{{⊕}_{e\in E}\rho_{e}}(G)\overset{˜}{\subseteq }e_{U}$. Thus, we have $G(e)\in \rho_{e}$ and z∈G(e) $\subseteq \left(Cl_{{⊕}_{e\in E}\rho_{e}}(G)\right)(e)\subseteq U$. Also, by Lemma 4.9 of [55], $\left(Cl_{{⊕}_{e\in E}\rho_{e}}(G)\right)(e)=Cl_{\rho_{e}}(G(e))$. Hence, $\left(Z,\rho_{e}\right)$ is almost *ω*-regular.

*Sufficiency*. Suppose that $\left(Z,\rho_{e}\right)$ is almost *ω*-regular for all e∈E. Let $e_{z}\in SP(Z,E)$ and $F\in \omega RO(Z,{⊕}_{e\in E}\rho_{e},E)$ such that $e_{z}\overset{˜}{\in }F$. Then by Theorem 2.17, $F(e)\in \omega RO(Z,\rho_{e})$. Since $\left(Z,\rho_{e}\right)$ is almost *ω*-regular and z∈F(e)∈ $\omega RO(Z,\rho_{e})$, then there exists $V\in \rho_{e}$ such that $z\in V\subseteq Cl_{\rho_{e}}(V)\subseteq F(e)$. Now, we have $e_{V}\in {⊕}_{e\in E}\rho_{e}$ and $e_{z}\overset{˜}{\in }e_{V}\overset{˜}{\subseteq }e_{Cl_{\rho_{e}}(V)}=Cl_{{⊕}_{e\in E}\rho_{e}}(e_{V})\overset{˜}{\subseteq }F$. It follows that $\left(Z,{⊕}_{e\in E}\rho_{e},E\right)$ is soft almost *ω*-regular. □

Corollary 3.26 *Let* (Z,ρ) *be a TS and E be any set of parameters. Then* (Z,τ(ρ),E) *is soft almost ω-regular if and only if* (Z,ρ) *is almost ω-regular.*

We end this section with a characterization of soft almost *ω*-regularity:

Theorem 3.27 *A STS* (Z,δ,E) *is soft almost ω-regular if and only if for each* $e_{z}\in SP(Z,E)$ *and each* F∈ωRO(Z,δ,E) *such that* $e_{z}\overset{˜}{\in }F$ *, there exists* M∈RO(Z,δ,E) *such that* $e_{z}\overset{˜}{\in }M\overset{˜}{\subseteq }Cl_{\delta }(M)\overset{˜}{\subseteq }F$ *.*

Proof *Necessity*. Suppose that (Z,δ,E) is soft almost *ω*-regular. Let $e_{z}\in SP(Z,E)$ and let F∈ωRO(Z,δ,E) such that $e_{z}\overset{˜}{\in }F$. Then there exists G∈δ such that $e_{z}\overset{˜}{\in }G\overset{˜}{\subseteq }Cl_{\delta }(G)\overset{˜}{\subseteq }F$. Thus, $e_{z}\overset{˜}{\in }Int_{\delta }\left(Cl_{\delta }(G)\right)\overset{˜}{\subseteq }F$. Since G∈δ, then $Int_{\delta }\left(Cl_{\delta }(G)\right)\in RO(Z,\delta ,E)$. Let $M=Int_{\delta }\left(Cl_{\delta }(G)\right)$. Then M∈RO(Z,δ,E) and

$$\begin{array}{ccc} e_{z}\overset{˜}{\in }M\; & \overset{˜}{\subseteq }\; & Cl_{\delta }(M)\\ \; & =\; & Cl_{\delta }\left(Int_{\delta }\left(Cl_{\delta }(G)\right)\right)\\ \; & \overset{˜}{\subseteq }\; & Cl_{\delta }\left(Cl_{\delta }(G)\right)\\ \; & =\; & Cl_{\delta }(G)\\ \; & \overset{˜}{\subseteq }\; & F\text{.} \end{array}$$

*Sufficiency*. Obvious. □

## 4 Soft nearly Lindelöfness

In this section, we characterize soft nearly Lindelöfness and improve several soft nearly Lindelöfness-related results by employing the concept of soft *ω*-regular open sets.

We start with the following characterization of soft nearly Lindelöf STSs:

Theorem 4.1 *For a STS,* (Z,δ,E) *the following are equivalent:*

*(a)* (Z,δ,E) *is soft nearly Lindelöf.*

*(b) Every soft cover of* $1_{E}$ *from* ωRO(Z,δ,E) *has a countable subcover.*

*(c) For every* M⊆ωRC(Z,δ,E) *with* $\overset{˜}{\cap_{M\in M}}M=0_{E}$ *, there exists a countable subfamily* $M_{1}\subseteq M$ *such that* $\overset{˜}{\cap_{M\in M_{1}}}M=0_{E}$ *.*

*(d) For every* M⊆ωRC(Z,δ,E) *with the countable soft intersection property,* $\overset{˜}{\cap_{M\in M}}M\neq 0_{E}$ *.*

Proof (a) ⟹ (b): Let G be a soft cover of $1_{E}$ from ωRO(Z,δ,E). For each $e_{z}\in SP(Z,E)$, there exists $G_{e_{z}}\in G$ such that $e_{z}\overset{˜}{\in }G_{e_{z}}$. Since $G_{e_{z}}\in \omega RO(Z,\delta ,E)$, then there exists $R_{e_{z}}\in RO(Z,\delta ,E)$ such that $e_{z}\overset{˜}{\in }R_{e_{z}}$ and $R_{e_{z}}-G_{e_{z}}\in CSS(Z,E)$. Since (Z,δ,E) is soft nearly Lindelöf and $\left\{R_{e_{z}}:e_{z}\in SP(Z,E)\right\}$ soft cover of $1_{E}$ from RO(Z,δ,E), there exists a countable subset H⊆SP(Z,E) such that $\left\{R_{e_{z}}:e_{z}\in H\right\}$ is a countable soft cover of G. We have

$$1_{E}=\overset{˜}{\cup_{e_{z}\in H}}\left(\left(R_{e_{z}}-G_{e_{z}}\right)\overset{˜}{\cup }G_{e_{z}}\right)=\left(\overset{˜}{\cup_{e_{z}\in H}}\left(R_{e_{z}}-G_{e_{z}}\right)\right)\overset{˜}{\cup }\;(\overset{˜}{\cup_{e_{z}\in H}}G_{e_{z}})\text{.}$$

Since *H* is countable and $R_{e_{z}}-G_{e_{z}}\in CSS(Z,E)$ for each $e_{z}\in H$, then $\overset{˜}{\cup_{e_{z}\in H}}\left(R_{e_{z}}-G_{e_{z}}\right)\in CSS(Z,E)$. Choose a countable subfamily $G_{1}$ of G such that $\overset{˜}{\cup_{e_{z}\in H}}\left(R_{e_{z}}-G_{e_{z}}\right)\overset{˜}{\subseteq }\overset{˜}{\cup_{G\in G_{1}}}G$. Set $G_{2}=\left\{G_{e_{z}}:e_{z}\in H\right\}\cup G_{1}$. Then $G_{2}$ is a countable subcover of G.

(b) ⟹ (c): Let M⊆ωRC(Z,δ,E) with $\overset{˜}{\cap_{M\in M}}M=0_{E}$. Then $\left\{1_{E}-M:M\in M\}\subseteq \omega RO(Z,\delta ,E\right)$ with $\overset{˜}{\cup_{M\in M}}\left(1_{E}-M\right)=1_{E}$. So by (b), there exists a countable subfamily $M_{1}\subseteq M$ such that $\overset{˜}{\cup_{M\in M_{1}}}\left(1_{E}-M\right)=1_{E}$. Hence, $\overset{˜}{\cap_{M\in M_{1}}}M=0_{E}$.

(c) ⟹ (d): Suppose to the contrary that there exists M⊆ωRC(Z,δ,E) with the countable soft intersection property such that $\overset{˜}{\cap_{M\in M}}M=0_{E}$. Then by (c), there exists a countable subfamily $M_{1}\subseteq M$ such that $\overset{˜}{\cap_{M\in M_{1}}}M=0_{E}$. Thus, M does not have the countable soft intersection property, which is a contradiction.

(d) ⟹ (a): Let G be a soft cover of $1_{E}$ from RO(Z,δ,E). Then $\left\{1_{E}-G:G\in G\right\}\subseteq RC(Z,\delta ,E)$ with $\overset{˜}{\cap_{G\in G}}\left(1_{E}-G\right)=0_{E}$. By Theorem 2.2, $\left\{1_{E}-G:G\in G\right\}\subseteq \omega RC(Z,\delta ,E)$. So, by (d), $\left\{1_{E}-G:G\in G\right\}$ does not have the countable soft intersection property. Thus, there exists a countable subset $G_{1}\subseteq G$ such that $\overset{˜}{\cap_{G\in G_{1}}}\left(1_{E}-G\right)=0_{E}$. Hence, $\overset{˜}{\cup_{G\in G_{1}}}G=1_{E}$. It follows that (Z,δ,E) is soft nearly Lindelöf. □

Theorem 4.2 *Let* (Z,δ,E) *be soft nearly Lindelöf STS. Then for each* K∈ωRC(Z,δ,E) *, K is soft nearly Lindelöf relative to* (Z,δ,E) *.*

Proof Let (Z,δ,E) be soft nearly Lindelöf and let K∈ωRC(Z,δ,E). Let S⊆RO(Z,δ,E) such that $K\overset{˜}{\subseteq }\overset{˜}{\cup_{S\in S}}S$. Since K∈ωRC(Z,δ,E), then $1_{E}-K\in \omega RO(Z,\delta ,E)$. Thus, for each $e_{z}\overset{˜}{\in }1_{E}-K$, there exists $R_{e_{z}}\in RO(Z,\delta ,E)$ such that $e_{z}\overset{˜}{\in }R_{e_{z}}$ and $R_{e_{z}}\overset{˜}{\cap }K=R_{e_{z}}-\left(1_{E}-K\right)\in CSS(Z,E)$. Since (Z,δ,E) is soft nearly Lindelöf, $\left\{S:S\in S\right\}\cup \left\{R_{e_{z}}:e_{z}\overset{˜}{\in }1_{E}-K\right\}\subseteq RO(Z,\delta ,E)$, and $1_{E}=\left(\overset{˜}{\cup_{S\in S}}S\right)\overset{˜}{\cup }\left(\overset{˜}{\cup_{e_{z}\overset{˜}{\in }1_{E}-K}}R_{e_{z}}\right)$, then there exist a countable subfamily $S_{1}\subseteq S$ and a countable set H⊆SP(Z,E) with $e_{z}\overset{˜}{\in }1_{E}-K$ for each $e_{z}\in H$ such that $1_{E}=\left(\overset{˜}{\cup_{S\in S_{1}}}S\right)\overset{˜}{\cup }\left(\overset{˜}{\cup_{e_{z}\overset{˜}{\in }H}}R_{e_{z}}\right)$. Since $\overset{˜}{\cup_{e_{z}\in H}}\left(R_{e_{z}}\overset{˜}{\cap }K\right)\in CSS(Z,E)$ and

$\begin{array}{ccc} \overset{˜}{\cup_{e_{z}\in H}}\left(R_{e_{z}}\overset{˜}{\cap }K\right)\; & =\; & \left(\overset{˜}{\cup_{e_{z}\in H}}R_{e_{z}}\right)\overset{˜}{\cap }K\\ \; & \overset{˜}{\subseteq }\; & K\\ \; & \overset{˜}{\subseteq }\; & \overset{˜}{\cup_{S\in S}}S\text{,} \end{array}$

then there exists a countable subfamily $S_{2}\subseteq S$ such that $\overset{˜}{\cup_{e_{z}\in H}}\left(R_{e_{z}}\overset{˜}{\cap }K\right)\overset{˜}{\subseteq }\overset{˜}{\cup_{S\in S_{2}}}S$. Hence, $S_{1}\cup S_{2}$ is a countable subfamily of S and $K\overset{˜}{\subseteq }\overset{˜}{\cup_{S\in S_{1}\cup S_{2}}}S$. It follows that *K* is soft nearly Lindelöf relative to (Z,δ,E). □

The following corollary is an immediate consequence of Theorem 2.2, Theorem 4.2:

Corollary 4.3 *Let* (Z,δ,E) *be soft nearly Lindelöf space. Then for each* K∈RC(Z,δ,E) *, K is soft nearly Lindelöf relative to* (Z,δ,E) *.*

Theorem 4.4 *Every soft semi ω-regular soft nearly Lindelöf STS is soft Lindelöf.*

Proof Let (Z,δ,E) be soft semi *ω*-regular and soft nearly Lindelöf. Let G be a soft cover of $1_{E}$ from *δ*. For each $e_{z}\in SP(Z,E)$, there exists $G_{e_{z}}\in G$ such that $e_{z}\overset{˜}{\in }G_{e_{z}}$. Since (Z,δ,E) is soft semi *ω*-regular, then for each $e_{z}\in SP(Z,E)$, there exists $M_{e_{z}}\in \omega RO(Z,\delta ,E)$ such that $e_{z}\overset{˜}{\in }M_{e_{z}}\overset{˜}{\subseteq }G_{e_{z}}$. Then $\left\{M_{e_{z}}:e_{z}\in SP(Z,E)\right\}$ is a soft cover of $1_{E}$ from ωRO(Z,δ,E). Since (Z,δ,E) is soft nearly Lindelöf, then by part (b) of Theorem 4.1, there exists a countable subset H⊆SP(Z,E) such that $\overset{˜}{\cup_{e_{z}\in H}}M_{e_{z}}=1_{E}$. Hence, $\left\{G_{e_{z}}:e_{z}\in H\right\}$ is a countable subfamily G such that $\left\{G_{e_{z}}:e_{z}\in H\right\}=1_{E}$. It follows that (Z,δ,E) is soft Lindelöf. □

Corollary 4.5 *Every soft semi regular soft nearly Lindelöf STS is soft Lindelöf.*

Corollary 4.6 *Every soft regular soft nearly Lindelöf STS is soft Lindelöf.*

Theorem 4.7 *Let* (Z,δ,E) *be soft almost regular and soft nearly Lindelöf space. Then for every* M,N∈RC(Z,δ,E) *such that* $M\cap N=0_{E}$ *, there exist* G,R∈δ *such that* $M\overset{˜}{\subseteq }G$ *,* $N\overset{˜}{\subseteq }R$ *, and* $G\overset{˜}{\cap }R=0_{E}$ *.*

Proof Since (Z,δ,E) is soft almost regular, for each $e_{z}\overset{˜}{\in }M$ by Theorem 3.4 (ii) of [50] there exists $S_{e_{z}}\in RO(Z,\delta ,E)$ such that $e_{z}\overset{˜}{\in }S_{e_{z}}$ and $Cl_{\delta }\left(S_{e_{z}}\right)\overset{˜}{\cap }N=0_{E}$. Since (Z,δ,E) is soft nearly Lindelöf space and $\left\{S_{e_{z}}:e_{z}\overset{˜}{\in }M\right\}\cup \left\{1_{E}-M\right\}$ is a soft cover of $1_{E}$ from RO(Z,δ,E), then there exists a countable set of soft points $t_{1},t_{2},...,t_{n},...$ such that $\left(\overset{˜}{\cup_{i\in N}}S_{t_{i}}\right)\overset{˜}{\cup }\left(1_{E}-M\right)=1_{E}$. It follows that $M\overset{˜}{\subseteq }\overset{˜}{\cup_{i\in N}}S_{t_{i}}$ and $Cl_{\delta }\left(S_{t_{i}}\right)\overset{˜}{\cap }N=0_{E}$ for all i∈N. Analogously there exists a family $\left\{T_{w_{i}}:i\in N\right\}\subseteq RO(Z,\delta ,E)$ such that $N\overset{˜}{\subseteq }\overset{˜}{\cup_{i\in N}}T_{w_{i}}$ and $Cl_{\delta }\left(T_{w_{i}}\right)\overset{˜}{\cap }M=0_{E}$ for all i∈N. For every n∈N, let $K_{n}=S_{t_{n}}-\overset{˜}{\cup_{i=1}^{n}}Cl_{\delta }(T_{w_{i}})$ and $H_{n}=T_{w_{n}}-\overset{˜}{\cup_{i=1}^{n}}Cl_{\delta }(S_{t_{i}})$. Let $G=\overset{˜}{\cup_{n\in N}}K_{n}$ and $R=\overset{˜}{\cup_{n\in N}}H_{n}$. Then G,R∈δ, $M\overset{˜}{\subseteq }G$, $N\overset{˜}{\subseteq }R$, and $G\overset{˜}{\cap }R=0_{E}$. □

Theorem 4.8 *Let* (Z,δ,E) *be soft almost ω-regular and soft nearly Lindelöf space. Then for every* M,N∈ωRC(Z,δ,E) *such that* $M\cap N=0_{E}$ *, there exist* G,R∈δ *such that* $M\overset{˜}{\subseteq }G$ *,* $N\overset{˜}{\subseteq }R$ *, and* $G\overset{˜}{\cap }R=0_{E}$ *.*

Proof Since (Z,δ,E) is soft almost *ω*-regular, for each $e_{z}\overset{˜}{\in }M$ by Theorem 3.27, there exists $S_{e_{z}}\in RO(Z,\delta ,E)$ such that $e_{z}\overset{˜}{\in }S_{e_{z}}$ and $Cl_{\delta }\left(S_{e_{z}}\right)\overset{˜}{\cap }N=0_{E}$. Since (Z,δ,E) is soft nearly Lindelöf space and $\left\{S_{e_{z}}:e_{z}\overset{˜}{\in }M\right\}\cup \left\{1_{E}-M\right\}$ is a soft cover of $1_{E}$ from ωRO(Z,δ,E), then by Part (b) of Theorem 4.1, there exists a countable set of soft points $t_{1},t_{2},...,t_{n},...$ such that $\left(\overset{˜}{\cup_{i\in N}}S_{t_{i}}\right)\overset{˜}{\cup }\left(1_{E}-M\right)=1_{E}$. It follows that $M\overset{˜}{\subseteq }\overset{˜}{\cup_{i\in N}}S_{t_{i}}$ and $Cl_{\delta }\left(S_{t_{i}}\right)\overset{˜}{\cap }N=0_{E}$ for all i∈N. Analogously there exists a family $\left\{T_{w_{i}}:i\in N\right\}\subseteq RO(Z,\delta ,E)$ such that $N\overset{˜}{\subseteq }\overset{˜}{\cup_{i\overset{˜}{\in }N}}T_{w_{i}}$ and $Cl_{\delta }\left(T_{w_{i}}\right)\overset{˜}{\cap }M=0_{E}$ for all i∈N. For every n∈N, let $K_{n}=S_{t_{n}}-\overset{˜}{\cup_{i=1}^{n}}Cl_{\delta }(T_{w_{i}})$ and $H_{n}=T_{w_{n}}-\overset{˜}{\cup_{i=1}^{n}}Cl_{\delta }(S_{t_{i}})$. Let $G=\overset{˜}{\cup_{n\in N}}K_{n}$ and $R=\overset{˜}{\cup_{n\in N}}H_{n}$. Then G,R∈δ, $M\overset{˜}{\subseteq }G$, $N\overset{˜}{\subseteq }R$, and $G\overset{˜}{\cap }R=0_{E}$. □

## 5 Conclusion

Soft *ω*-regular open sets have been introduced. It is proved that the collection of soft *ω*-regular open sets forms a soft topology that lies strictly between the classes of soft regular open sets and soft *ω*-open sets. Soft semi *ω*-regularity and soft almost *ω*-regularity have been also introduced as two new independent variants of soft regularity via soft *ω*-regular open sets. In addition, the relationships between soft topology and classical (parametric) topology have been revealed. Finally, nearly Lindelöfness has been characterized, and several results related to soft nearly Lindelöfness have been improved. In future studies, the following topics could be considered: 1) define new classes of soft functions using soft *ω*-regular open sets; 2) investigate the behavior of soft semi *ω*-regular and soft almost *ω*-regular STSs in the context of product STSs; and 3) characterize some types of soft Menger spaces using soft *ω*-regular open sets.

## Declarations

### Author contribution statement

Samer Al Ghour: Conceived and designed the experiments; Analyzed and interpreted the data; Contributed reagents, materials, analysis tools or data; Wrote the paper.

### Funding statement

This research did not receive any specific grant from funding agencies in the public, commercial, or not-for-profit sectors.

### Data availability statement

No data was used for the research described in the article.

### Declaration of interests statement

The authors declare no conflict of interest.

### Additional information

No additional information is available for this paper.
